# Noninvasive Assessment of Aortic Pulse Wave Velocity by the Brachial Occlusion-Cuff Technique: Comparative Study

**DOI:** 10.3390/s19163467

**Published:** 2019-08-08

**Authors:** Vratislav Fabian, Lukas Matera, Kristyna Bayerova, Jan Havlik, Vaclav Kremen, Jan Pudil, Pavol Sajgalik, David Zemanek

**Affiliations:** 1 Department of Physics, Faculty of Electrical Engineering, Czech Technical University in Prague, 166 27 Prague, Czech Republic; 2 2nd Department of Internal Medicine—Cardiology and Angiology of General University Hospital and 1st Medical Faculty of Charles University, 128 08 Prague, Czech Republic; 3 Department of Circuit Theory, Faculty of Electrical Engineering, Czech Technical University in Prague, 166 27 Prague, Czech Republic; 4 Czech Institute of Informatics, Robotics, and Cybernetics, Czech Technical University in Prague, 160 00 Prague, Czech Republic; 5 Department of Cardiovascular Diseases, Mayo Clinic, Rochester, MN 559 05, USA

**Keywords:** arterial stiffness, pulse wave velocity, suprasystolic blood pressure, single-cuff

## Abstract

Cardiovascular diseases are one of most frequent cause of morbidity and mortality in the world. There is an emerging need for integrated, non-invasive, and easy-to-use clinical tools to assess accurately cardiovascular system primarily in the preventative medicine. We present a novel design for a non-invasive pulse wave velocity (PWV) assessment method integrated in a single brachial blood pressure monitor allowing for up to 100 times more sensitive recording of the pressure pulsations based on a brachial occlusion-cuff (suprasystolic) principle. The monitor prototype with built-in proprietary method was validated with a gold standard reference technique SphygmoCor VX device. The blood pressure and PWV were assessed on twenty-five healthy individuals (9 women, age (37 ± 13) years) in a supine position at rest by a brachial cuff blood pressure monitor prototype, and immediately re-tested using a gold standard method. PWV using our BP monitor was (6.67 ± 0.96) m/s compared to PWV determined by SphygmoCor VX (6.15 ± 1.01) m/s. The correlation between methods using a Pearson’s correlation coefficient was r = 0.88 (*p* < 0.001). The study demonstrates the feasibility of using a single brachial cuff build-in technique for the assessment of the arterial stiffness from a single ambulatory blood pressure assessment.

## 1. Introduction

Cardiovascular diseases are one of the most important causes of morbidity and mortality in the world. Known risk factors include smoking, arterial hypertension, hyperlipidemia, diabetes, metabolic syndrome, etc. Although cardiovascular mortality has declined in developed countries as a result of better treatment and prevention in recent decades, the identification of individuals with higher risk is very important and can prevent severe consequences [1]. Early preventive intervention can lead to delaying or even averting their manifestations. Besides the blood pressure (BP), arterial stiffness is one of options to assess the risks since it is associated with pathophysiology of arterial hypertension and is often evaluated by measuring pulse wave velocity (PWV). In particular, the aortic stiffness is one of determinant of the absolute central versus peripheral BP (e.g., arm) difference; it has been recognized as a significant factor of cardiovascular health, and as an important index for prediction of arterial hypertension and a high BP [2]. In general, arterial stiffness quantifies mechanical properties of the arterial wall is an independent predictor for fatal and non-fatal cardiovascular events in hypertensive patients. Ultimately, it provides an added value above and beyond traditional risk factors, which was recognized in a number of studies [3,4]. Although the relationship between aortic stiffness and events is continuous, a threshold value is 10 m/s in middle-aged hypertensive patients can differentiate those with higher cardiovascular risk [5].

The most common equation which describes the arterial stiffness is the relationship between change in pressure (ΔP) and change in volume (ΔV). Structurally, elastin, collagen and smooth muscles are the main components of the large conduit arteries and this composition is changing during ageing, which is closely associated with developing the arterial stiffness and hypertension.

Aortic higher PWV represents a consequence of arterial stiffness. The PWV is related to the wall stiffness, vessel geometry and blood density. These relationships are described by Moens-Korteweg equation [6,7]. A carotid-femoral PWV is often considered as a gold standard for a non-invasive measurement of PWV [8]. Nowadays, it is the only method, which is accurate enough to be considered as a diagnostic method and describes the PWV in the aorta. The PWV is calculated as a ratio of a distance between carotid and femoral artery and pulse transit time between these two points.

There are few non-invasive methods for the evaluation of arterial stiffness and PWV. In the current clinical practice, the SphygmoCor technology (AtCor Medical, Australia), and VaSera (Fukuda, Japan) devices are the most frequently used. The SphygmoCor technology uses applanation tonometry for obtaining the pulse wave from the carotid and femoral arteries and estimates PWV from the delay between pulse waves on the carotid and femoral arteries with respect to ECG [9,10,11,12]. The VaSera device uses the four-cuff oscillometric system with the ECG recording and an amorphous pulse wave sensor placed on the carotid and the femoral arteries. The device estimates several parameters correlated with arterial stiffness, especially the PWV and CAVI index [13,14,15,16,17]. These devices are relatively expensive and requires trained technician for accurate results, which is disadvantageous for implementing in the wider general internal, and preventative offices. Another method for PWV estimation is Arteriograph (Tensiomed, Hungary). The Arteriograph technology uses a different approach than the SphygmoCor technology and VaSera devices in principle. The Arteriograph technology uses an analysis of oscillometric pulsations detected in the brachial artery for indirect estimation of PWV by the difference in time between the beginning of the first (forward) and the second (reflected) pulse wave. This delay corresponds to the distance between the jugulum and the symphysis. The method does not use ECG recording [9]. Since the Arteriograph uses a single cuff for data acquisition, the practical use is remarkably higher, however the signal processing requires sophisticated signal processing for obtaining required parameters.

For the purpose of this study we utilized a newly developed proprietary principle [18] for non-invasive recording of highly accurate raw signal of blood pressure waveform requiring minimal filtering without introducing a potential bias in the PVW assessment. This technique stands out as a single brachial cuff technique and a viable platform for development an easy-to-operate complex hemodynamic monitor, which could deliver information about peripheral BP, PWV, and estimate central blood pressure during a one short patient visit. As such, this technique will be well suitable in the preventive and family care medicine. We hypothesize that the below proposed concept of integrated hemodynamic monitor will overcome practical disadvantages of gold standard methods (requiring more than one signal from the human body for PWV analysis) and yet delivering reliable and validated results in a user-friendly design.

## 2. Materials and Methods

### 2.1. Amended Brachial Occlusion-Cuff Technique

For the purpose of the study, we have developed a prototype of BP monitor using unique hardware and software features. In principle, the device is based on settle detection of pressure pulsations from brachial arm cuff, which is pressurized (35–40) mm Hg above systolic pressure. This pressure is called a ‘suprasystolic’ or ‘stop flow’. During this pressure, the brachial artery is completely occluded and the propagating pulse waves, created by contraction of the left ventricle and the ejection of blood into the aorta, are completely transmitted through arm cuff to pressure sensors.

**Figure 1:**
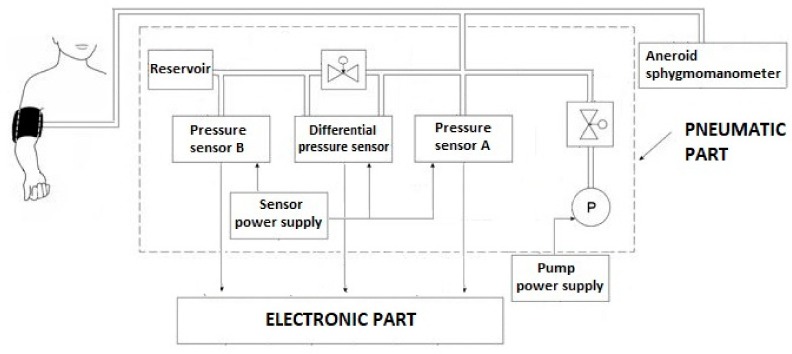
Pneumatic part of experimental device [18].

Very sensitive differential pressure sensor (range ± 3.8 mm Hg) enables to detect even very weak pressure pulsations. The block diagram of the pneumatic part of the device is shown in Figure 1. Upon reaching the suprasystolic pressure, the closing valve is closed, and the reservoir is pneumatically separated from the cuff. Thus, at the differential sensor output we see only superimposed pressure pulses from the cuff. Conventional devices use gauge pressure sensors with a range of at least 300 mm Hg to detect pressure pulses. With this differential pressure sensor is our method 40 times more sensitive compared to these conventional devices. It eliminates the disadvantages of existing devices, in particular the necessity of using a compensation filter or derived models [18].

**Figure 2:**
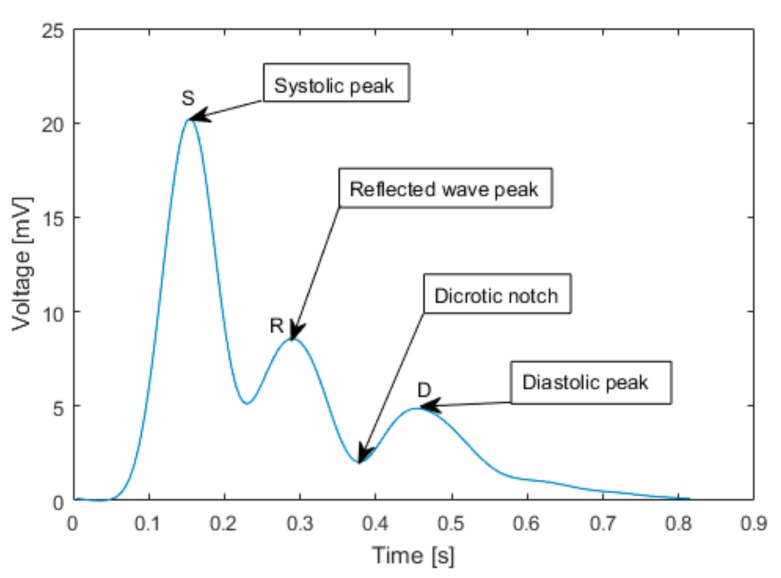
The typical pulse wave acquired from the left brachial artery by the arm cuff inflated to suprasystolic pressure. Systolic peak (S) corresponds to contraction of the left ventricle, Reflected wave peak (R) corresponds to reflected wave from iliac bifurcation and Diastolic peak (D) corresponds to the delayed reflected wave from the lower body.

The typical pulse wave obtained by averaging of several acquired cardiac cycles is shown in Figure 2. During the data evaluation, the signal of each cardiac cycle is divided into segments separated by significant peaks. The first one is a systolic peak (S), which corresponds to contraction of the left ventricle. The consequent peaks detected by differential pressure sensor are systolic peak reflected from iliac bifurcation (R) and a delayed diastolic peak reflected from the lower body (D).

The time interval between S and R is ΔT and it is the time, which takes the pulse wave to travel from the arch of aorta to iliac bifurcation and back to brachial artery, where is the pulse wave detected. The iliac bifurcation is known as a main source of the reflection of the systolic peak.

The distance, which traveled the measured pulse wave, was determined as a distance between the jugulum and the half of distance between umbilicus and symphysis.

The developed device consists of 3 pressure sensors (two gauge pressure sensors, one differential pressure sensor), an electric pump, a brachial arm cuff, two valves and electronic circuits.

The analog outputs from pressure sensors are amplified and digitized by the BIOPAC MP36 system, which uses 24-bit sigma-delta A/D converter (SNR: > 89 dB min Tested at lowest Gain at 1000 s/s with grounded front end, CMRR: 110 dB minimum at 50/60 Hz) with a sampling frequency 200 Hz. The BIOPAC MP36 system was used because it meets the high medical regulations for medical devices.

### 2.2. Measurement Protocol

Measurements were performed in cooperation with 2nd Department of Internal Medicine—Cardiology and Angiology of General University Hospital and 1st Medical Faculty of Charles University (Prague, Czech Republic). All subjects signed the informed consent approved by the local ethical committee.

Before the beginning of the measurement, the physician measured the blood pressure of the subject in a sitting position. After that, the subject moved to the supine position and after 10 min of rest the first phase of the measurement started. The PWV of the subject was first measured with SphygmoCor VX according methodology [5], i.e., it was taken two measurements and if the difference between the two measurements was more than 0.5 m/s, it was performed a third measurement and the median value was used. That took approximately twenty minutes and after that, we continued with measurement using the prototype of the device. All SphygmoCor measurements were performed by the same physician.

The second phase of the measurement began with inflation of the arm cuff (standard OMRON CM2 cuff) in the pneumatic part of the measurement system to the suprasystolic pressure by an electrical pump. As was mentioned, the suprasystolic pressure is about (35–40) mm Hg above systolic pressure. The arm cuff was inflated about 5 mm Hg even more because of the stabilization the pressure in the pneumatic part and also because of the pressure drop, which was about 2 mm Hg per minute. After the stabilization of the pressure in the circuit, the mechanical valve was closed and the pressure pulsations from brachial arm cuff were sensed by the differential pressure sensor.

After twenty seconds of the suprasystolic pressure pulsations measurement the mechanical valve was turned back to open state and the valve was released the pressure out of the arm cuff and the whole pneumatic circuit by electromagnetic decompression. The pressure in the cuff was recorded, and values were continuously stored in the memory of the device (sampling frequency F_s_ = 200 Hz). Similarly, we took two measurements and if the difference between the two measurements was more than 0.5 m/s we performed a third measurement and used the median value. All measurements were instrumented by an identical qualified technician.

As a distance for PWV evaluation, we used a distance ref. equation defined by:

DIST_PWV_ = |JUG-UMB| + |UMB-SYM|/2 (1)

where |JUG-UMB| is a distance between jugulum and umbilicus and |UMB-SYM| is a distance between umbilicus and symphysis.

### 2.3. Study Population

All participants were healthy volunteers from hospital staff. Each volunteer was familiar with the measurement and gave informed consent to participate in the research. A total of 25 participants were measured during the study (9 women). Table 1 displays demographics of the study participants. Exclusion criteria were: second level of obesity, defined by body mass index (BMI) > 35 kg·m^−2^, systolic blood pressure (SBP) > 180 mm Hg or diastolic blood pressure (DBP) > 120 mm Hg, which means a hypertensive crisis, the history of any cardiovascular diseases, a taking of any regular medication or subjects with an arm circumference that was outside the range of (23–32) cm. After the measurements, participants with the reporting signs of any cardiac arrhythmias were also excluded from the study (one man, AF) and also participants, whose data was not possible to evaluate because of erroneous readings from the SphygmoCor VX (one subject), or from the experimental device (one subject). One woman was not included in the study because of her pregnancy, which was found afterwards. Nobody had diabetes and only two were smokers. All subjects gave their informed consent for inclusion before they participated in the study. The study was conducted in accordance with the Declaration of Helsinki, and the protocol was approved by the Ethics Committee of General University Hospital, Prague, Czech Republic (Project identification code: 89/16 Grant VES 2017 AZV VFN), 23 June 2016.

### 2.4. Data Evaluation

PWV was determined using a custom GUI software in MATLAB Inc. In the first part of algorithm, we choose only an effective part of measured signals. The algorithm is applied only to the parts of the data, where suprasystolic pulse waves were measured. The data where the pneumatic leaks caused distortions were filtered by algorithm for baseline wander removing (polynomial fitting and designed high-pass FIR filter). High-pass FIR filter with cut-off frequency of 0.5 Hz designed by Hamming window was used to remove isoline by polynomial fitting for every suprasystolic pulsation. To remove high frequency noise, caused by breathing, moving artifacts, electromagnetic artifacts with higher frequencies, and the frequency 50 Hz, caused by line noise interference, we used a low-pass FIR filter with cut-off frequency 20 Hz.

The determination of PWV during suprasystolic pressure depends on the right detections of systolic peaks and reflected wave peaks (Figure 2), caused by reflection in aortic bifurcation, which is supposed to be the main source of wave reflection [19]. We based our detection algorithm on the first and second order difference combined with thresholding. The identified potential, systolic as well as reflected peaks, was then compared with criteria, which was used for the determine a PWV. The time difference between two suprasystolic waves had to be in the interval of HR (30 < HR < 200) beats/min. As a secondary criterion, each value of PWV that exceeded a physiological interval (3 < PWV < 15) m/s was considered as an artefact and was excluded from the results [20]. The PWVs, which values were different than (MEAN ± 1.96·STD) were also excluded and the final PWV was to be determined at least from 5 suprasystolic pulse waves.

### 2.5. Statistical Analysis

The statistical analysis was performed in MATLAB Inc. software statistical toolboxes. The data were divided into quantitative and qualitative groups. The quantitative data were summarized by their MEAN ± STD values (Table 1) the qualitative by percentage representations in the dataset. The error analysis is in the detail described in the discussion. The comparative analysis included Bland-Altman plots, regression analysis, Pearson correlation analysis and Lin’s concordance correlation coefficient. Bland-Altman plots shows a graphical representation of the comparison between two methods by analysis of their means and differences and show a potential sign of correlation [21], which was determined by Pearson’s correlation coefficient. To confirm relationship between measurements, we also calculated Lin’s concordance correlation coefficient [22]. To define linear association and its regression coefficients we calculated a linear regression analysis. All analyses were two-tailed and associated *p* values of less than 5% were taken as indication of statistical significance.

## 3. Results

Patients’ demographics are described in Table 1. From a total number of 25 participants, 21 were included in the analysis. The reasons for exclusion of 4 participants are described in the study population section of the methods. The mean age of 21 subjects (8 women) was 37 ± 13 years (21 to 66 years). Regarding to one BP, there was only one hypertensive patient and according to BMI, only two of them had first class obesity and higher.

The multivariate linear regression (*p* < 0.001) confirmed the hypothesis about PWV as an age dependent parameter. Other characteristics did not have so strong correlation in this study sample.

**Figure 3:**
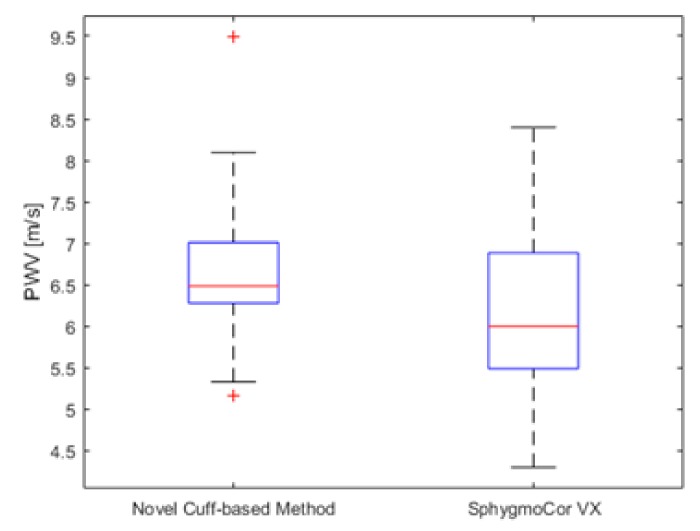
Device comparison of PWV measurements.

The mean PWV measured by novel cuff-based method was (6.67 ± 0.96) m/s compared to SphygmoCor VX (6.15 ± 1.0) m/s. The mean difference between methods was (0.61 ± 0.35) m/s. According to Artery society guidelines [23] that describes the process of validating new device for PWV measurement, a new device is acceptable, if the mean difference with standard deviation of results between the validated device and the ground truth device is not more than (1.0 ± 1.5) m/s. Figure 3 graphically displays a non-significant difference (*p* > 0.05) between measurements of both devices.

**Figure 4:**
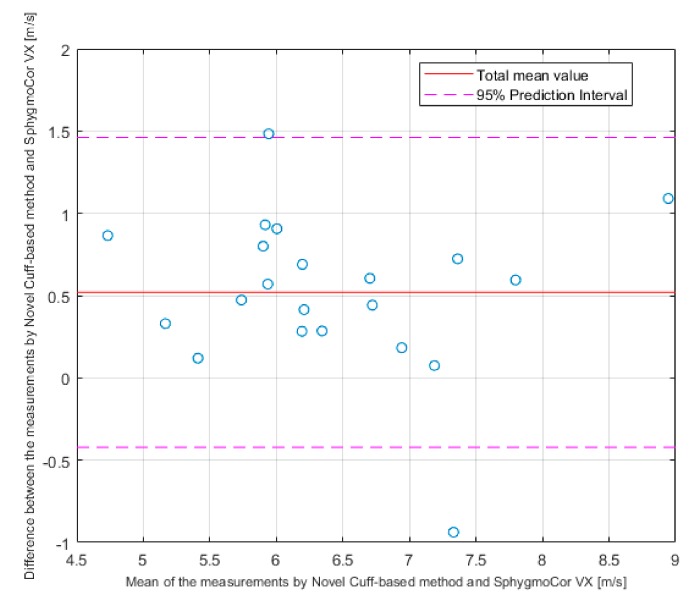
Bland-Altman plot.

Pearson’s correlation coefficient r = 0.88 (*p* < 0.001) and Lin’s concordance correlation coefficient r = 0.77 confirmed a relationship between the novel cuff-based method and SphygmoCor VX, which determines cfPWV a gold standard (ground truth) in PWV measurement. Bland-Altman plot (Figure 4) graphically displays a comparison of methods in our dataset and shows the measurements that differ from the mean value more than ± 1.96·STD (95% confidence interval). In our case, there were only two values outside this confidence interval (10%).

## 4. Discussion

The aim of the study was to present a novel, proprietary method for ad non-invasive measurement of PWV based on the cuff occlusion and to test how the method compares to ground truth using a gold standard measurement device SphygmoCor VX. The vision of this novel method is to provide a non-invasive, fast, easy, and yet precise determination of hemodynamic parameters without a need of having trained personnel performing the measurement. SphygmoCor allows to measure hemodynamic parameters that are used to assess static and dynamic variables of the cardiovascular system. One of a few hemodynamic parameters, which could be determined by SphygmoCor VX is carotid-femoral PWV (cfPWV) [24]. The cfPWV is traditionally used as a gold standard for PWV measurements and is considered as one of the most important predictors of arterial stiffening, an important feature of cardiovascular system. Arterial stiffness is recognized as a significant factor of cardiovascular health and potentially as an important index of prediction of hypertension and high pulse pressure. SphygmoCor VX was chosen as a reference for comparison with our method because its widely use in the Europe, where cfPWV is considered as a key parameter for arterial stiffness prediction, unlike in Asia regions, where is more common to predict arterial stiffness by brachial-ankle PWV (baPWV).

SphygmoCor VX is based on the applanation tonometry principle with simultaneous measurement of synchronous ECG. It works as a time reference for determination of cfPWV and results in sequential-based measurement, which, amongst others, results in longer estimation time. The measurement should be provided by trained physician or technician and has to follow strict protocol to obtain reliable results. Our method removes these limitations of having ECG, trained specialist, and strict protocol. This new method is standardized, cuff-based measurement with a fully automatic control of the pressure in the brachial cuff. The measurement is very short, repeatable and can be carried easy in clinic or even in the home environment, and thus suits well for screening and disease prevention

**Figure 5:**
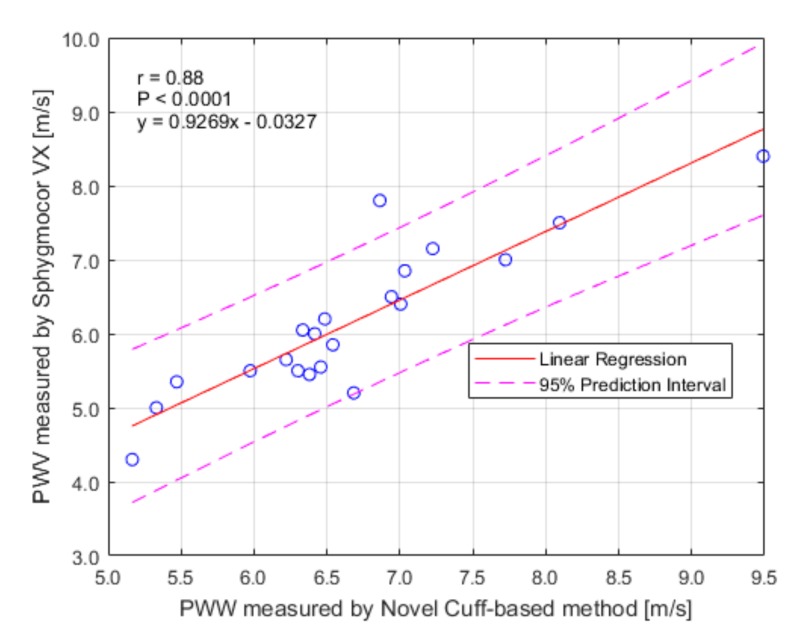
Linear regression.

The results have shown a high correlation between both methods (r = 0.88, *p* < 0.001). All the readings, except one, lay in the ± 1.96·STD of the mean value. The analysis of the differences between values (0.61 ± 0.35) m/s described by B-A plot shown the trend of the shift. According to the artery society, this result can be taken as an acceptable and close to an excellent result [23]. Based on these results we can say that the method systematically measures slightly higher PWV values than the ground truth SphygmoCor VX device. This systematic error is given by methodologically imperfect measurement of the distance described above. As was previously described in the study [25], it is possible to use umbilicus as a mark for the AB. Our method used a distance that was taken closer to symphysis (Equation (1)) to try to be in the middle of the 80% population interval for the generation of the main reflected wave. This ‘higher’ landmark resulted in systematic estimation of distance shorter than actually is and it caused a difference of means (0.52 m/s). This conclusion was also confirmed by the linear regression analysis (Figure 5), where the shift was also observed.

Despite of the pros of the method, there are several limitations that needs to be considered. Most of them are caused by random and systematic errors during the measurement itself. First limitation is the precision of a manual measurement that determines the aortic bifurcation (AB), which is considered as a main source of the forward wave reflection. The precision of AB determination and its distance from the jugulum is one of the most important parameters in the computation of the PWV. According to the study [25], it is possible to get close to the real position of the AB, but only in 80% of the population is AB and close surroundings taken as the main source of the reflection [19]. In the rest of them, the source of the reflection is moved closer to the femoral sites, which makes the distance longer. This also depends on the shape of the aorta. There are few factors that change the shape and subsequently the distance of traveling of the pulse wave. However, our study population had wide range of height (from 165 cm to 198 cm), and the results show that the correlation of results from both devices is still good across all subjects of the group. This is a clear limitation of our method in face of methods with ECG synchronization and sequential measurements that get the distance precise because of the known positions of the pulse wave acquisition sensors. The other important systematic error during the data recording is caused by a resolution of tape measure, sampling frequency, and leakages in the pneumatic circuit and comes with the data evaluation by developed algorithms. These algorithms do filtering, averaging, and peak detection to find maximums of the forward and the reflected wave in the signal to estimate the times of propagation. Sometimes a various shapes and amplitudes of every reflected waves lead in a total dampening of the reflected wave so there is no maximum but only an inflection point in the forward primary wave and seemed to be a very challenging and difficult to detect automatically. To deliver a high-quality automated monitor, further studies need to be focused on the optimization of the pneumatic system, precise determination of aortic bifurcation distance, and development of the algorithms for the accurate recognition of the primary and reflected waves in situation of contra-phase coupling.

## 5. Conclusions

Our results confirmed that the novel design of single brachial-cuff technique generating high fidelity signal compares very closely with the current gold standard method SphygmoCor VX for PVW assessment. Results have also shown that PWV is an age-dependent parameter, where the PWV is increasing with the age because of the loss of the elasticity of aortic walls and their calcification. Since the clinical need is to recognize the alterations of cardiovascular system prior developing organ complications, the presented technique can be used in the home monitoring and primary care setting.

A proprietary hardware solution allows for building the presented design in a standard non-invasive brachial cuff blood pressure monitor. The simplicity of use, yet ability to provide consistent results strongly supports feasibility of the proposed solution in preventive care and can be translated into clinical practice.

## Figures and Tables

**Table 1:** Study participants.

Subject No.	Sex	Age (Years)	Height (cm)	Weight (kg)	BMI (kg·m^−2^)	BMI Classification	SBP (mm Hg)	DBP (mm Hg)
1	F	23	165	60	22.0	Normal weight	127	75
2	M	21	193	86	23.1	Normal weight	151	72
3	M	66	178	94	29.7	Overweight	167	86
4	M	36	188	86	24.3	Normal weight	151	87
5	F	50	170	69	23.9	Normal weight	127	82
6	F	54	173	64	21.4	Normal weight	117	65
7	F	53	167	63	22.6	Normal weight	151	94
8	M	34	190	80	22.2	Normal weight	127	80
9	M	32	180	92	28.4	Overweight	122	63
10	F	40	174	73	24.1	Normal weight	125	74
11	M	46	183	105	31.4	Obesity Class 1	122	67
12	M	25	180	75	23.1	Normal weight	126	66
13	F	23	173	67	22.4	Normal weight	122	74
14	F	42	172	62	21.0	Normal weight	118	70
15	F	39	165	62	22.8	Normal weight	116	72
16	M	35	174	71	23.5	Normal weight	112	68
17	M	21	184	108	31.9	Obesity Class 1	128	68
18	M	21	180	75	23.1	Normal weight	106	68
19	M	41	192	86	23.3	Normal weight	118	68
20	M	29	198	110	28.1	Overweight	126	72
21	M	53	176	75	24.2	Normal weight	118	72
Mean ± STD		37.3 ± 12.6	178.8 ± 9.2	79.2 ± 15.2	24.6 ± 3.2		127.5 ± 14.7	73.5 ± 7.9
